# Chiropractic identity, role and future: a survey of North American chiropractic students

**DOI:** 10.1186/s12998-014-0048-1

**Published:** 2015-02-02

**Authors:** Jordan A Gliedt, Cheryl Hawk, Michelle Anderson, Kashif Ahmad, Dinah Bunn, Jerrilyn Cambron, Brian Gleberzon, John Hart, Anupama Kizhakkeveettil, Stephen M Perle, Michael Ramcharan, Stephanie Sullivan, Liang Zhang

**Affiliations:** Private Practice, 725 S. Dobson Rd, Suite 100, Chandler, AZ 85224 USA; Logan University College of Chiropractic, 1851 Schoettler Rd, Chesterfield, MO 63017 USA; Northwestern University of Health Sciences, 2501 W. 84th St, Bloomington, MN 55431 USA; National University of Health Sciences, 200 E. Roosevelt Rd, Lombard, IL 60148 USA; Canadian Memorial College of Chiropractic, 6100 Leslie St, Toronto, Ontario Canada; Sherman College of Chiropractic, 2020 Springfield Rd, Boiling Springs, SC 29316 USA; Southern California University of Health Sciences, 16200 E. Amber Valley Dr., Whittier, CA 90604 USA; University of Bridgeport, Bridgeport, CT 06604 USA; Texas Chiropractic College, 5912 Spencer Highway, Pasadena, TX 77505 USA; Life University, 1269 Barclay Circle, Marietta, GA 30060 USA; Palmer College of Chiropractic - Florida, 4777 City Center Parkway, Port Orange, FL 32129 USA

**Keywords:** Chiropractic, Cross-sectional survey

## Abstract

**Background:**

The literature pertaining to chiropractic students’ opinions with respect to the desired future status of the chiropractic physician is limited and is an appropriate topic worthy of study. A previous pilot study was performed at a single chiropractic college. This current study is an expansion of this pilot project to collect data from chiropractic students enrolled in colleges throughout North America.

**Objective:**

The purpose of this study is to investigate North American chiropractic students’ opinions concerning professional identity, role and future.

**Methods:**

A 23-item cross-sectional electronic questionnaire was developed. A total of 7,455 chiropractic students from 12 North American English-speaking chiropractic colleges were invited to complete the survey. Survey items encompassed demographics, evidence-based practice, chiropractic identity and setting, and scope of practice. Data were collected and descriptive statistical analysis was performed.

**Results:**

A total of 1,247 (16.7% response rate) questionnaires were electronically submitted. Most respondents agreed (34.8%) or strongly agreed (52.2%) that it is important for chiropractors to be educated in evidence-based practice. A majority agreed (35.6%) or strongly agreed (25.8%) the emphasis of chiropractic intervention is to eliminate vertebral subluxations/vertebral subluxation complexes. A large number of respondents (55.2%) were not in favor of expanding the scope of the chiropractic profession to include prescribing medications with appropriate advanced training. Most respondents estimated that chiropractors should be considered mainstream health care practitioners (69.1%). Several respondents (46.8%) think that chiropractic research should focus on the physiological mechanisms of chiropractic adjustments.

**Conclusion:**

The chiropractic students in this study showed a preference for participating in mainstream health care, report an exposure to evidence-based practice, and desire to hold to traditional chiropractic theories and practices. The majority of students would like to see an emphasis on correction of vertebral subluxation, while a larger percent found it is important to learn about evidence-based practice. These two key points may seem contradictory, suggesting cognitive dissonance. Or perhaps some students want to hold on to traditional theory (e.g., subluxation-centered practice) while recognizing the need for further research to fully explore these theories. Further research on this topic is needed.

**Electronic supplementary material:**

The online version of this article (doi:10.1186/s12998-014-0048-1) contains supplementary material, which is available to authorized users.

## Introduction

The last thirty years in health care have brought about many changes in thoughts and practice ideologies. One of these recent trends is an emphasis on cost-effective treatments and interprofessional collaboration [1-3]. Additional changes in health care over this time have included an increase in medical specialization and sub-specialization, the concept and implementation of evidence-based practice, and a greater acceptance of complementary and alternative medicine (CAM) therapies in mainstream medicine. Amid all of these transformations and shifts in the health care arena, a primary spine care specialist role has not been established. The current state of spinal care has been classified as a “supermarket approach” consisting of multiple practitioners including primary care providers, chiropractic physicians, acupuncturists, physical therapists, physiatrists, orthopedic surgeons, neurosurgeons, massage therapists, and naturopathic physicians with multiple treatment philosophies, high salesmanship and little interprofessional communication [4]. Chiropractic physicians possess many attributes that would be required of a primary spine care practitioner, and with specific modifications in education and practice, chiropractors may be in a position to make a relatively lateral transition to occupy this role [4]. As factions of the chiropractic profession are establishing a pathway for chiropractors to assume an evidence-based primary spine care practitioner role integrated in mainstream health care, it has been asserted this may not be the route many field providers desire to pursue [5]. Although most professions may have internal factions with conflicting viewpoints, such factions within chiropractic are particularly contentious [6]. According to McGregor et al., progressing toward a collaborative focus will demand a more visible appreciation of the professional strata that exist, and the mutual goals that exist between them [1]. Because chiropractic students represent the future of the profession, examining their views on professional identity might provide insight into the future of the profession. However, to date, there has been little research done among this population, with only one related specifically to this topic; this study, in fact, served as the pilot study for the current project [7]. The aim of this investigation was to survey chiropractic students’ opinions about chiropractic identity, role and future. Results may yield an insight into future practitioners’ perspective about the future of the profession, thus aiding in chiropractic’s progression. This study may further act as a catalyst for future studies directed to current practicing chiropractors.

## Methods

### Survey development

A 23-item survey instrument (Additional file 1) was developed by the lead investigators at Logan University, based on the pilot survey previously used by the principal investigator among chiropractic college students at one university [7]. The survey instrument was reviewed by all remaining investigators and feedback was provided about the content, with subsequent revisions made. Survey items encompassed demographics, evidence-based practice, chiropractic identity and setting, and scope of practice. The first 8 survey items collected demographic information that included participants’ current chiropractic institution in which they are enrolled, age, sex, current enrollment status, education and degrees achieved prior to enrollment, and student chiropractic organization affiliations. The enrollment status question was constructed to standardize the various institutions’ use of semesters, trimesters, and quarters into 1st, 2nd or 3rd year. The remaining 15 survey items (9–23) explored participants’ opinions concerning evidence-based practice, chiropractic identity and setting, and scope of practice. Of the final 15 items, 11 were constructed in 5-point Likert scale with the following ratings: 1 = Strongly Agree; 2 = Agree; 3 = Neutral; 4 = Disagree; 5 = Strongly Disagree.

### Sample population

Initially, the research directors of all 19 accredited North American chiropractic colleges were invited by email to participate by administering the survey at their institution and contributing to the analysis and reporting of results. Of these 19 institutions, 12 participated (63%), each with at least one designated on-site representative to administer the survey. The participating institutions were:

- Canadian Memorial Chiropractic College
- Logan University
- National University of Health Sciences
- Northwestern Health Sciences University
- Palmer College of Chiropractic – Davenport campus
- Palmer College of Chiropractic – Florida campus
- Palmer College of Chiropractic – West campus
- Sherman College of Chiropractic
- Southern California University of Health Sciences
- Texas Chiropractic College
- University of Bridgeport

### Eligibility criteria

Students enrolled in an accredited Doctor of Chiropractic (DC) degree program at the time of survey administration at a participating institution were eligible. Students enrolled in a bachelor, master or non-chiropractic doctoral degree program without concurrently being enrolled in a DC program, or those enrolled part-time in a DC program, were ineligible.

### Survey administration

An anonymous cross-sectional survey to identify chiropractic students’ opinions regarding chiropractic identity, role and future was administered among 12 North American English-speaking chiropractic colleges. The lead institution’s (Logan University) Institutional Review Board (IRB) approved this study and all participating colleges that required IRB submission of the project obtained approval from their respective institutions. A notice of participant consent was included at the beginning of the electronic survey. Participation in this study was voluntary and no compensation was provided to students for participating in this study. The electronic survey was conducted through Survey Monkey. Logan University’s Survey Monkey Platinum account ensured HIPAA compliance at the highest level to all survey participants. Survey Monkey is electronic survey administrations system in which questions appear on the screen and scroll to the next item after being answered. Respondents were able to return to questions and change their responses at any time until they submitted the questionnaire; at that time it was no longer accessible by that respondent. We used a setting which only allows one survey response per IP address, in order to avoid duplication. Responses were then downloaded into an Excel file.

The study’s program coordinator uploaded the survey instrument into Survey Monkey. A representative at each participating institution disseminated student recruitment invitations and the survey instrument to all eligible chiropractic students via email between November 2013 and April 2014. However, specific survey recruitment varied depending upon policies and procedures in place at each institution. Due to logistical difficulties and also to avoid duplicate responses, reminders were not sent to the students.

### Data analysis

Results were downloaded from Survey Monkey directly into SPSS (v.22). Descriptive statistics were computed. Frequencies were computed for all variables, including those items with Likert scale categories (strongly agree, agree, neutral, disagree, strongly disagree and missing). Responses were compared for each item, stratified by year in program. Using a Chi square test, an alpha level of .05 was used to indicate statistically significant differences among responses by year in program.

## Results

A total of 1,247 surveys were completed. Among the 12 institutions participating, response rates varied from 38.0% to 6.1%, with a mean response of 16.72. Table 1 details the response rates by institution.

**Table 1:** **Response rates by institution**

**Institution**	**n distributed**	**n completed**	**Response rate (%)**
1	677	257	38.0
5	434	99	22.8
3	485	104	21.4
10	744	137	18.4
2	602	106	17.6
6	310	49	15.8
9	234	36	15.4
8	1830	263	14.4
12	920	93	10.1
4	148	14	9.5
7	725	53	7.3
11	346	21	6.1
**Total**	**7455**	**1247**	**16.7**

Respondent demographics are reported in Table 2. The majority of respondents were between the ages of 18–25 years (50.9%) and male (53.6%). However, when comparing the sample by year in program, the proportion of men was less for earlier years in the program (50.3% for year 1, 53.2% for year 2, and 58.6% for year 3). Most respondents (83.4%) reported obtaining a bachelor’s degree prior to enrollment in chiropractic college, with few respondents indicating further education beyond a bachelor’s degree. The majority of respondents (62.1%) reported belonging to neither Student International Chiropractic Association (SICA) nor Student American Chiropractic Association (SACA). Respondents’ current status in the Doctor of Chiropractic (DC) program were well represented throughout each year of enrollment with 394 respondents (31.6%) reporting a status of 1st year, 400 (32.1%) as 2nd year, and 446 (35.8%) as 3rd year. A significant number of 3rd year respondents (86.0%) reported having completed a course in evidence-based practice in their chiropractic curriculum.

**Table 2:** **Respondent demographics**

**Sex**	n	%
Male	668	53.6
Female	565	45.3
Missing	14	1.1
**Age**		
18-25 years	635	50.9
26-35 years	489	39.2
36-45 years	91	7.3
46-55 years	20	1.6
55 years and older	7	.6
Missing	5	.4
**Highest level of education prior to enrollment**		
Associate degree	110	8.8
Bachelor degree	1040	83.4
MA/MS/MPH degree	63	5.1
Doctoral degree (Ph.D, EdD, etc.)	16	1.3
Missing	18	1.4
**Professional degrees prior to enrollment**		
MD/DO from USA	4	.3
MD/DO from other country	6	.5
Other health professional degree (RN, PT, etc.)	111	8.9
Other professional degree (JD, etc.)	59	4.7
None	1036	83.1
Missing	31	2.5
**Membership in chiropractic organizations**		
Student International Chiropractic Association	94	7.5
Student American Chiropractic Association	326	26.1
Both	45	3.6
Neither	775	62.1
Missing	7	.6
**Current enrollment status in DC program**		
1st year	394	31.6
2nd year	400	32.1
3rd year	446	35.8
Missing	7	.6
Total	1247	100.0
**Report having completed a course in evidence-based practice in chiropractic program**	782	63.5
Year 1	137	35.0
Year 2	265	66.4
Year 3	380	86.0

Responses to statements regarding evidence-based practice, identity and settings, and scope of practice are outlined in Table 3. Most respondents agreed (34.8%) or strongly agreed (52.2%) that it is important for chiropractors to be educated in evidence-based practice. Approximately half (51.9%) of the respondents strongly agreed (21.6%) or agreed (30.3%) that contemporary and evolving scientific evidence is more important than traditional chiropractic theory. Nearly half (45.8%) of respondents strongly agreed (17.4%) or agreed (28.4%) that it is important for chiropractors to hold strongly to traditional chiropractic theories and practices. Many respondents strongly agreed (39.5%) or agreed (29.7%) that it is important for the progression of the chiropractic profession to include clinical chiropractic training internships and post-graduate residencies in integrative medical settings. A majority agreed (35.6%) or strongly agreed (25.8%) the emphasis of chiropractic intervention is to eliminate vertebral subluxations/vertebral subluxation complexes; however very few respondents strongly agreed (6.5%) or agreed (6.6%) that chiropractic intervention should consist of chiropractic adjustment only. A large number of respondents (55.2%) were not in favor of expanding the scope of the chiropractic profession to include prescribing medications with appropriate advanced training.

**Table 3:** **Respondents’ agreement with statements about practice and identity**

	**SA (%)**	**A (%)**	**N (%)**	**D (%)**	**SD (%)**	**M (%)**
**Evidence based practice**						
It is important for chiropractors to be educated in evidence based practice.	52.2	34.8	6.0	1.3	0.2	5.5
It is appropriate to allow for updating and enrichment of chiropractic theories based on current scientific advancements.	47.8	39.1	5.0	2.1	.8	5.3
Contemporary and evolving scientific evidence is more important than traditional chiropractic theory	21.6	30.3	25.2	13.6	3.8	5.6
It is important for chiropractors to hold strongly to traditional chiropractic theories and practices.	17.4	28.4	23.5	19.0	6.2	5.5
**Identity and setting**						
Chiropractic providers should maintain portal of entry (direct access) status.	57.2	26.2	9.1	1.3	.5	5.7
Inclusion of clinical chiropractic training internships and post-graduate residencies in integrative medical settings is important to the progression of the chiropractic profession.	39.5	29.7	12.7	7.2	5.5	5.5
**Scope of practice**						
Emphasis of chiropractic intervention is to eliminate vertebral subluxations/vertebral subluxation complexes.	25.8	35.6	15.2	10.3	6.9	6.1
The primary purpose of the chiropractic examination is to detect vertebral subluxations.	20.7	23.9	15.8	22.0	12.0	5.6
The chiropractic profession should expand its scope of practice to include prescribing of medication, with appropriate advanced training	12.9	13.2	13.2	18.8	36.4	5.5
It is appropriate for the chiropractic profession to distinguish and promote two separate subgroups of broad scope (providing manual and other non-drug procedures) and limited scope (providing subluxation correction only).	10.3	20.7	26.6	21.6	15.6	5.3
Chiropractic intervention should consist of chiropractic adjustment only.	6.5	6.6	8.9	34.2	38.5	5.4

SA = Strongly Agree; A = Agree; N = Neutral; D = Disagree; SD = Strongly Disagree; M = Missing.

Table 4 shows additional responses relating to identity and setting, and scope of practice. Most respondents deemed that chiropractors should be considered mainstream health care practitioners (69.1%). The largest proportion of respondents (46.9%) deemed the most important chiropractic practice paradigm is one that focuses on primary spine/musculoskeletal care; 25.5% thought it should be primary care; and 15.4% that it should focus on subluxation correction only. The highest proportion of participants (46.8%) reported that chiropractic researchers should focus future efforts primarily on physiological mechanisms of chiropractic adjustments.

**Table 4:** **Respondents’ opinions, by topic**

**Doctors of chiropractic should be considered**	n	%
Complementary/alternative health practitioners	312	25.0
Mainstream health care practitioners	862	69.1
Missing	73	5.9
**The most important practice paradigm for the chiropractic profession is**		
Subluxation correction only focus	192	15.4
Primary spine/musculoskeletal care physician	585	46.9
General/Primary care physician	318	25.5
Other	81	6.5
Missing	71	5.7
**The most important setting for chiropractic health care is**		
Integrated settings with other health care disciplines including allopathic medicine	354	28.4
Integrated settings with alternative medicine practitioners only	51	4.1
Alone or with other DC’s, without integration with any other health care disciplines	102	8.2
Any/all of the above	670	53.7
Missing	70	5.6
**Chiropractic researchers should focus future efforts primarily on**		
Physiological mechanisms of chiropractic adjustments	584	46.8
Outcomes/cost-effectiveness of chiropractic care	349	28.0
Outcomes/cost-effectiveness of integrative care models	240	19.2
Missing	74	5.9

Responses were additionally analyzed by year of respondents’ current DC program status. Table 5 illustrates respondents’ responses to statements about practice and identity, by topic and year in DC program. Items included in Table 5 were only those in which there was a statistically significant difference among years in program. Nearly all respondents agreed that chiropractic providers should maintain portal of entry status (84.7% 1st year, 90.1% 2nd year, and 90.1% 3rd year). Respondents tended to disagree with the idea of expansion of chiropractic scope to include prescribing medications more so in 2nd (61.6%) and 3rd (60.2%) year respondents compared to 1st year (53.5%). Respondents in their 3rd year (18.7%) agreed that chiropractic intervention should consist of chiropractic adjustment only more so than 1st (11.3%) and 2nd (10.9%) year respondents, although most respondents disagreed with this statement regardless of year in DC program.

**Table 5:** Respondents’ agreement with statements about practice and identity, by topic and year in program*

	**A (%)**	**N (%)**	**D (%)**
**Identity and setting**			
Chiropractic providers should maintain portal of entry (direct access) status.			
Year 1	84.7	14.0	1.3
Year 2	90.1	8.3	1.6
Year 3	90.1	7.3	2.6
Inclusion of clinical chiropractic training internships and post-graduate residencies in integrative medical settings is important to the progression of the chiropractic profession.			
Year 1	74.3	17.9	7.8
Year 2	73.0	13.1	13.9
Year 3	72.5	9.4	18.1
**Scope of practice**			
Emphasis of chiropractic intervention is to eliminate vertebral subluxations/vertebral subluxation complexes.			
Year 1	67.6	17.8	14.6
Year 2	67.2	17.1	15.7
Year 3	61.8	14.2	23.9
The chiropractic profession should expand its scope of practice to include prescribing of medication, with appropriate advanced training.			
Year 1	33.1	13.4	53.5
Year 2	22.7	15.7	61.6
Year 3	26.9	12.9	60.2
It is appropriate for the chiropractic profession to distinguish and promote two separate subgroups of broad scope (providing manual and other non-drug procedures) and limited scope (providing subluxation correction only).			
Year 1	37.8	29.5	32.7
Year 2	28.4	28.1	43.5
Year 3	32.1	26.9	41.0
Chiropractic intervention should consist of chiropractic adjustment only.			
Year 1	11.3	11.0	77.7
Year 2	10.9	6.4	82.7
Year 3	18.7	10.5	70.7

NOTE—all the above differed significantly.

*Items are only included if the differences among year in program responses were significant at the p < .05 level (Chi square test).

A = agree; N = neutral; D = disagree.

Table 6 shows respondents’ opinions by year in DC program. Items included in Table 6 were those in which there was a statistically significant difference among years in program. A greater percentage of respondents viewed primary spine/musculoskeletal care as the most important practice paradigm for the chiropractic profession in 3rd year (54.2%) respondents compared to 1st (48.7%) and 2nd (45.7%) year respondents. Interestingly, students in favor of a subluxation correction only focus paradigm tended to increase in 3rd year (21.0%) respondents compared to 1st (15.6%) and 2 year (12.0%) respondents. Respondents tended to view physiological mechanisms of chiropractic adjustments as the most important focus for chiropractic researchers less as they progressed in the DC program (54.7% 1st year, 49.1% 2nd year, and 46.4% 3rd year). Additionally, respondents in favor of outcomes/cost-effectiveness of chiropractic care as a primary chiropractic research focus increased by percentage as respondents progressed in the DC program (23.8% 1st year, 32.3% 2nd year, and 32.7% 3rd year).

**Table 6:** Respondents’ opinions by year in program*

	**Yr 1 (%)**	**Yr 2 (%)**	**Yr 3 (%)**	**Total (%)**
**Doctors of chiropractic should be considered**				
Complementary/alternative health practitioners	25.3	23.0	30.8	26.6
Mainstream health care practitioners	74.7	77.0	69.2	73.4
**The most important practice paradigm for the chiropractic profession is**				
Subluxation correction only focus	15.6	12.0	21.0	16.4
Primary spine/musculoskeletal care physician	48.7	45.7	54.2	49.7
General/Primary care physician	29.6	34.3	18.4	27.0
Other	6.2	8.0	6.4	6.8
**The most important setting for chiropractic health care is**				
Integrated settings with other health care disciplines including allopathic medicine	25.5	34.2	30.5	30.1
Integrated settings with alternative medicine practitioners only	5.6	2.4	5.0	4.3
Alone or with other DC’s, without integration with any other health care disciplines	7.8	6.1	11.8	8.7
Any/all of the above	61.1	57.3	52.7	56.9
**Chiropractic researchers should focus future efforts primarily on**				
Physiological mechanisms of chiropractic adjustments	54.7	49.1	46.4	49.9
Outcomes/cost-effectiveness of chiropractic care	23.8	32.3	32.7	29.8
Outcomes/cost-effectiveness of integrative care models	21.4	18.7	20.9	20.4

NOTE—all the above differed significantly.

*Items are only included if the differences among year in program responses were significant at the p < .05 level (Chi square test).

By the third year of chiropractic education, most (86.0%) students reported taking a class in evidence-based practice. A majority of respondents expressed an appreciation for education in evidence-based practice (87.0%), the importance of contemporary scientific evidence (51.9%), and the appropriateness of enriching chiropractic theories based on current scientific advancements (86.9%). Comparison of respondents based on year in DC program showed little variance in issues related to opinions on evidence-based practice.

## Discussion

This study suggests that North American chiropractic students appreciate evidence-based practice, have a desire to participate in mainstream health care, be considered mainstream practitioners, concentrate in musculoskeletal/primary spine care, and are not in favor of expanding the chiropractic scope of practice to include prescribing medications. Results of this study also suggest that chiropractic students desire to hold onto aspects of traditional chiropractic theories and practices such as emphasizing subluxation/vertebral subluxation complex as part of the evaluation and treatment of patients. However, a majority of the students also expressed the desire for researching physiological mechanisms of chiropractic adjustments. This may indicate a trend that future chiropractors want to maintain traditional principles while pursuing a more scientific understanding of those principles. According to the Institute for Alternative Futures (IAF) Chiropractic 2025 report, general recommendations were set out for the chiropractic community to pursue including: 1. Integrate chiropractic into health care systems and integrative care models, 2. Increase research, including why chiropractic adjustments produce cost-effective outcomes, why they often show positive non-musculoskeletal effects, and how they influence gene expression and self-healing, 3. Continue to strive for high standards of practice, including promoting the use of evidence-based guidelines [8]. Results of this survey suggest that chiropractic students’ ideologies mirror these aforementioned recommendations of IAF which might suggest positive strides toward continued chiropractic acceptance and growth.

The opinions and perceptions of these students may indicate future implications for the chiropractic profession. According to IAF, chiropractic has remained largely secluded from integrated health care delivery systems. [8] It is expected that 30-85% of health care will be comprised of these integrated systems. [8] Inclusion in these models, which students in this study supported, may yield greater public acceptance and utilization of chiropractic services [8]. This may additionally require modifications and shifts in traditional emphasis in chiropractic curriculum such as increased exposure to integrative settings, emphasis in primary spine care management in an integrative setting, accelerating research, and emphasis on evidence-based guidelines.

The majority of students (61.4%) would like to see an emphasis on correction of vertebral subluxation in chiropractic practice. In addition, a larger percent (87.0%) said it is important to learn about evidence-based practice. These two key points may seem contradictory, suggesting cognitive dissonance. Or perhaps some students want to hold on to traditional theory (e.g., subluxation-centered practice) while recognizing the need for further research to fully explore these theories, as suggested by the data that showed 86.9% of the sample said that it is appropriate to update chiropractic theories based on scientific advancements. More research on this topic is needed.

As health care continues to evolve and take strides toward the establishment of collaborative multidisciplinary teams and a shift from fee-for-service reimbursements to bundled payments, risk sharing, and/or capitation [8], chiropractors must decide their identity and role in relation to this new model of health care. The chiropractic profession has been handcuffed with internal discord [9,10] and multiple strata within the profession exist. Current chiropractic students will play a role in contributing to choosing the path the profession chooses to pursue in this time of health care reorganization and this study shows the current climate of North American chiropractic students’ ideologies in relation to professional identity and role. Future studies should focus on assessing practicing chiropractic physicians, and this study can provide a springboard for future investigations.

## Limitations

This study, much like other studies of similar nature, had limitations. The low response rate (16.7%) was the greatest limitation; a 2008 study reported online surveys to have an average response rate of 33% [11]. There is consequently likely to be a response bias. Repetitive survey distributions or reminders may have provided an increase in response rate. The survey may not have been representative due to response bias; we were only able to assess response bias in terms of gender and SACA membership. Nearly half of respondents (45.3% of total respondents and 49.7% of 1st year respondents) of this study were female. This represents a higher percentage of females in the chiropractic profession than previously reported [12]. In 2009 only 22.4% of the chiropractic profession was made up of females, unlike the typical chiropractic patient which is estimated to be approximately 60% female, compared to 48.8% of female medical graduates that year [12]. This high proportion of women students might suggest that women were overrepresented in our sample.

Approximately one fourth (26.1%) of respondents reported membership in Student American Chiropractic Association (SACA). Recent reporting of enrollment in United States (US) based chiropractic colleges has been described as 9,946 students in 2010 [8]. As of June 2014, SACA membership is 3,429 students (Hall LC, ACA Director of Membership Operations/SACA Liaison, personal communication Jun 3, 2014), representing approximately 35% of US based chiropractic students, which is comparable to the results of this study. This provides evidence of the representativeness of our sample in terms of SACA membership.

A substantial proportion of respondents reported a “neutral” stance for multiple survey items. As with all surveys constructed with a middle response option, potential sources of response error exist. [13] A vast majority of respondents reported having a course in evidence-based practice; however responses to this survey item, along with responses to all items of the survey instrument, may be incomplete due to the bias of the sample available to this study.

Our sample in this study may not be representative of all North American chiropractic colleges in terms of approach to chiropractic philosophy, although this study did include colleges with diverse philosophical approaches [8].

Results of this study represent only a subset of North American students at a particular time in history. Particularly given that there were only 63% of North American chiropractic colleges that participated in this study, and there are more chiropractic colleges located outside of North America, results of this study may not be representative of chiropractic students as a whole.

## Conclusion

Results of this study indicate that North American chiropractic students show a preference for participation in integrative health care, have been exposed to evidence-based practice concepts, and appreciate a practice paradigm focusing on musculoskeletal/primary spine care. However students indicate a desire to hold strongly to traditional chiropractic theories and practices, including emphasis in vertebral subluxation/vertebral subluxation complex. Students in our study display certain ideologies that are in alignment with recommendations for the accelerated progression of chiropractic’s future.

## Additional file

Additional file 1:
**Survey instrument.**

## References

[1] McGregor M, Puhl AA, Reinhart C, Injeyan HS, Soave D (2014). Differentiating intraprofessional attitudes toward paradigms in health care delivery among chiropractic factions: results from a randomly sampled survey. BMC Complement Altern Med.

[2] Gaboury I, Bujold M, Boon H, Moher D (2009). Interprofessional collaboration within Canadian integrative healthcare clinics: key components. Soc Sci Med.

[3] Kreitzer MJ, Kligler B, Meeker WC (2009). Health professions education and integrative healthcare. Explor.

[4] Murphy DR, Justice BD, Paskowski IC, Perle SM, Schneider MJ (2011). The establishment of a primary spine care practitioner and its benefits to health care reform in the united states. Chiropr Man Ther.

[5] Villanueva-Russell Y (2011). Caught in the crosshairs: identity and cultural authority within chiropractic. Soc Sci Med.

[6] McGregor-Triano M: Jurisdictional Control of Conservative Spine Care: Chiropractic Versus Medicine. The University of Texas at Dallas: University of Texas at Dallas; 2006.

[7] Gliedt JA, Briggs S, Williams JS, Smith DP, Blampied J (2012). Background, expectations and beliefs of a chiropractic student population: a cross-sectional survey. J Chiropr Educ.

[8] Institute for Alternative Futures (2013). Chiropractic 2025: Divergent Futures.

[9] Gliedt JA: Finding a common ground in chiropractic: the key to progression. *Top Integr Health Care* 2011, 2(4).

[10] Kaptchuck TJ, Eisenberg DM (1998). Chiropractic: origins, controversies, and contributions. Arch Intern Med.

[11] Nulty D (2008). The adequacy of response rates to online and paper surveys: what can be done?. Assess Eval High Educ.

[12] Johnson CD, Green BN (2012). Diversity in the chiropractic profession: preparing for 2050. J Chiropr Educ.

[13] Sturgis P, Roberts C, Smith P (2014). Middle alternatives revisited: how the neither/nor response acts as a way of saying “i don’t know”?. Sociol Methods Res.

