# Supplementary material for: Chiropractic identity, role and future: a survey of North American chiropractic students
Source: Chiropr Man Therap. 2015 Feb 2;23:4. doi: 10.1186/s12998-014-0048-1 (PMC4313466; doi:10.1186/s12998-014-0048-1)
Supplement: Additional file 1: — Survey instrument. [file 12998_2014_48_MOESM1_ESM.docx]

Chiropractic student assessment of identity, role and future.

**Please mark only one response for each question.**

**Background**

1. Age:
   1. 18-25 years
   2. 26-35 years
   3. 36-45 years
   4. 46-55 years
   5. Older than 55 years
2. Sex:
   1. Male
   2. Female
3. The following best describes my current enrollment status in the doctor of chiropractic degree program:
   1. 1st-3rd trimester / 1st-4th quarter / 1^st^ -2^nd^ semester
   2. 4^th^-6^th^ trimester / 5^th^-8^th^ quarter / 3^rd^ -5^th^ semester
   3. 7^th^-10^th^ trimester / 9^th^-13^th^ quarter / 6^th^ -8^th^ semester
4. Highest level of education achieved prior to enrollment in an accredited doctor of chiropractic degree program:
   1. Associate degree
   2. Bachelor degree
   3. MA/MS/MPH degree
   4. Doctoral degree (PhD, EdD, etc.)
5. Health professional/other professional degrees achieved prior to enrollment in an accredited doctor of chiropractic degree program:
   1. MD/DO from USA medical/osteopathic school
   2. MD/DO from any other country
   3. Other health professional degree (RN, PT, etc.)
   4. Other professional degree (JD, etc.)
   5. None
6. I am a member of:
   1. Student International Chiropractic Association
   2. Student American Chiropractic Association
   3. Both
   4. Neither
7. I have completed a course in evidence-based practice in my doctor of chiropractic degree program:
   1. Yes
   2. No

**Chiropractic Identity, Role and Future**

1. It is important for chiropractors to hold strongly to the traditional chiropractic theory that adjusting the spine corrects “dis-ease.”
   1. Strongly Agree
   2. Agree
   3. Neutral
   4. Disagree
   5. Strongly Disagree
2. It is important for chiropractors to be educated in evidence-based practice:
   1. Strongly Agree
   2. Agree
   3. Neutral
   4. Disagree
   5. Strongly Disagree
3. Contemporary and evolving scientific evidence is more important than traditional chiropractic principles:
   1. Strongly Agree
   2. Agree
   3. Neutral
   4. Disagree
   5. Strongly Disagree
4. It is appropriate to allow for updating and enrichment of chiropractic theories based on current scientific advancements:
   1. Strongly Agree
   2. Agree
   3. Neutral
   4. Disagree
   5. Strongly Disagree
5. Doctors of chiropractic should be considered:

a. Complementary/alternative health care practitioners

1. Mainstream health care practitioners
2. The most appropriate practice paradigm for the chiropractic profession is:
    a. Subluxation correction only focus
    b. Primary spine/musculoskeletal care physician
    c. General/primary care physician
    d. Other
3. The most appropriate setting for chiropractic health care is:
   1. Integrative settings with other health care disciplines including allopathic medicine
   2. Integrative settings with alternative medicine practitioners only
   3. Alone or with other DC’s, without integration with any other health care disciplines
   4. Any/all of the above
4. Inclusion of clinical chiropractic training internships and post-graduate residencies in integrative medical settings is important to the progression of the chiropractic profession:
   1. Strongly Agree
   2. Agree
   3. Neutral
   4. Disagree
   5. Strongly Disagree
5. Chiropractic providers should maintain portal of entry (direct access) status:
   1. Strongly Agree
   2. Agree
   3. Neutral
   4. Disagree
   5. Strongly Disagree
6. The primary purpose of the chiropractic examination is to detect vertebral subluxations:
   1. Strongly Agree
   2. Agree
   3. Neutral
   4. Disagree
   5. Strongly Disagree
7. Emphasis of the chiropractic intervention is to eliminate vertebral subluxations/vertebral subluxation complexes:
   1. Strongly Agree
   2. Agree
   3. Neutral
   4. Disagree
   5. Strongly Disagree
8. Chiropractic intervention should consist of chiropractic adjustment only:
   1. Strongly Agree
   2. Agree
   3. Neutral
   4. Disagree
   5. Strongly Disagree
9. The chiropractic profession should expand its scope of practice to include prescribing of medication, with appropriate advanced training:
   1. Strongly Agree
   2. Agree
   3. Neutral
   4. Disagree
   5. Strongly Disagree
10. Chiropractic researchers should focus future efforts primarily on:
    1. Physiological mechanisms of chiropractic adjustments
    2. Outcomes/cost-effectiveness of chiropractic care
    3. Outcomes/cost-effectiveness of integrative care models
11. It is appropriate for the chiropractic profession to distinguish and promote two separate subgroups of broad scope (providing manual and other non-drug procedures) and limited scope (providing subluxation correction only).
    1. Strongly Agree
    2. Agree
    3. Neutral
    4. Disagree
    5. Strongly Disagree
